# Medical Association of Alabama

**Published:** 1883-05-20

**Authors:** 


### *MEDICAL ASSOCIATION OF ALABAMA.*

This body met at Birmingham on the 10th of April. There was a good turn out and a number of interesting papers read.

The officers elected for the ensuing year are as follows:

Dr. Frank Tipton, of Selma, and Dr. S. M. Hogan, of Union Springs, Vice-Presidents; Dr. T. A. Means, Secretary, and Dr. W. C. Jackson, Treasurer.

Resolutions were adopted condemning the New York Code of Ethics; also all journals and colleges which adopt or in any degree favor said Code.

